# Screening of *BRCA1* variants c.190T>C, 1307delT, g.5331G>A and c.2612C>T in breast cancer patients from North India

**DOI:** 10.1590/1678-4685-GMB-2019-0014

**Published:** 2020-05-20

**Authors:** Akeen Kour, Vasudha Sambyal, Kamlesh Guleria, Neeti Rajan Singh, Manjit Singh Uppal, Mridu Manjari, Meena Sudan

**Affiliations:** 1 Guru Nanak Dev University, Department of Human Genetics, Human Cytogenetics Laboratory, Amritsar, Punjab, India.; 2 Sri Guru Ram Das Institute of Medical Sciences and Research, Vallah,Department of Surgery, Amritsar, Punjab, India.; 3 Sri Guru Ram Das Institute of Medical Sciences and Research, Vallah,Department of Pathology, Amritsar, Punjab, India.; 4 Sri Guru Ram Das Institute of Medical Sciences and Research, Vallah, Department of Radiotherapy, Amritsar, Punjab, India.

**Keywords:** BRCA1, variants, breast cancer, North India

## Abstract

The polymorphic variants of *BRCA1*, which lead to amino acid substitutions, have a known pathogenic role in breast cancer. The present study investigated in North Indian breast cancer patients the association of risk with four reported pathogenic variants of *BRCA1*: c.190T>C (p.Cys64Arg), 1307delT, g.5331G>A (p.G1738R) and c.2612C>T (p.Pro871Leu). Genotyping was done by PCR-RFLP method in 255 clinically confirmed breast cancer patients and 255 age and gender matched healthy individuals. For c.190T>C, 1307delT and g.5331G>A, all the patients and controls had the wild-type genotype indicating no association with breast cancer risk. For c.2612C>T polymorphism, the frequency of the CC, CT, and TT genotypes was 14.5 vs 15.7%, 59.6 vs 53.7% and 25.9 vs 30.6% in breast cancer patients and controls respectively. The frequency of heterozygotes (CT genotype) was higher in cases than controls but the difference was not statistically significant. Genetic model analysis showed no association of the four analyzed *BRCA1* variants with breast cancer risk with any model. The studied variants were not associated with the risk of breast cancer in Punjab, North west India, suggesting a need for further screening of other *BRCA1* variants. It is the first reported study on these 4 variants from India.

## Introduction

Breast cancer is the most common cancer in the world (Ghoncheh *et al.*, 2016). In India, according to the National Cancer Registry Programme (NCRP 2012-2014), incidence of breast cancer ranks amongst the top in the following individual registries across India: Delhi (41.0%), Chennai (37.9%), Bangalore (34.4%) and Thiruvananthapuram district (33.7%). In the state of Punjab, the highest incidence rate of breast cancer has been reported in districts of Bathinda (37.3%), followed by Mohali (34.3%), Patiala (32.5%) and Faridkot (31.5%) (NCRP 2012-2013), with increasing frequency of sporadic breast cancer being reported from Amritsar (Batra *et al.*, 2010).

One of the well-studied genes that has been associated with breast cancer, *BRCA1* (MIM 113705), is located at the chromosomal position 17q21 and contains 24 exons (Miki *et al.*, 1994). It encodes a multi-domain protein BRCA1, which has four major domains: a RING domain, the BRCA1 serine domain and two BRCA1 C terminus (BRCT) domains (Clark *et al.*, 2012). BRCA1 interacts with a variety of other proteins to carry out multiple functions at the cellular level, like controlling the cell cycle, DNA damage repair, regulation of transcription, replication, recombination and chromatin hierarchical control (Parvin, 2004).

Mutations in *BRCA1* and *BRCA2* genes are the most susceptible causes amongst the genetic risk factors that play a crucial role in familial breast cancer (Naga, 2011). A frequency of 2.9-24% of *BRCA1/2* mutations has been reported in familial breast cancer patients of South India (Vaidyanathan *et al.*, 2009). However, the reported frequency of mutations in *BRCA2* is remarkably lower than in *BRCA1* in India (Kim and Choi, 2013).

Populations of Caucasian and Indoscythian mixed racial ancestry inhabit the state of Punjab in North India (Bhasin *et al.*, 1992). Therefore, the *BRCA1* variants were chosen for the study considering their prevalence in similar Caucasian and South Asian populations in other parts of the world. The four variants of *BRCA1* [c.190T>C (p.Cys64Arg), 1307delT, g.5331G>A (p.G1738R) and c.2612C>T (p.Pro871Leu)] were investigated in the current study. The variant c.2612C>T (p.Pro871Leu) is a non-synonymous polymorphism of *BRCA1* that leads to the substitution of proline by leucine at position 871, a region where recombinase RAD51 interacts with BRCA1 to aid homologous recombination (Miao *et al.*, 2017). The T allele has been reported to show an association with breast cancer risk in Chinese populations (Xu *et al.*, 2018) though some studies have reported no significant association between the p.Pro871Leu variant of *BRCA1* and breast cancer risk. The 1307delT variant of *BRCA1*, present in codon 396 of exon 11, causes a frameshift mutation that introduces a premature stop codon at amino acid residue 409 and leads to protein truncation. It was reported in the South Indian population (Gajalakshmi *et al.*, 2007). The c.1907T>C variant of *BRCA1* is a missense mutation present in exon 5 (Cys64Arg). Molecular modelling indicated that the substitution of cysteine with an arginine probably disturbs the structure of the BRCA1 RING finger domain, which is responsible for the interaction with BARD1 and essential for the tumor-suppressor activity of the BRCA1-BARD1 complex (Karami and Mehdipour*,* 2013). g.5331G>A (p.G1738R) is a missense variant of *BRCA1* that affects the binding affinity of the protein by destabilizing it (Williams *et al.*, 2003; Glover, 2006). It has been reported as a pathogenic mutation (Chenevix-Trench *et al.*, 2006; Karchin *et al.*, 2007).

Although a few common variants of *BRCA1* have been repeatedly reported from South India, the dissimilarity between the populations of North India: Caucasians, Scythians and Indo-Europeans (Bhasin *et al.*, 1992; Reich *et al.*, 2009) and South India: Dravidian (Reich *et al.*, 2009), led us to consider studying the variants in North India. Thus, the current study aimed to screen the Punjabi population from Amritsar and adjoining regions of Punjab, North India, for the four variants of *BRCA1* [c.190T>C (p.Cys64Arg), 1307delT, g.5331G>A (p.G1738R) and c.2612C>T (p.Pro871Leu)] to investigate their association with breast cancer risk. Identifying *BRCA1* polymorphisms that are associated with breast cancer risk can also be further explored for involvement in the therapy response of patients. *BRCA1* c.190T>C (p.Cys64Arg) and g.5331G>A (p.G1738R) have never been reported from India in any of the studies so far. Also, these four variants together have not been investigated in Indian patients in any previous study.

## Subjects and Methods

### Study subjects

In this case-control study, patients were selected from a tertiary care hospital, Sri Guru Ram Das Institute of Medical Sciences and Research, Vallah, Amritsar, Punjab. The majority of the patients (81%) belonged to the Majha region of Punjab, i.e., to the Amritsar district (34%) and its adjoining districts of Gurdaspur (32%) and Tarn Taran (15%). The rest of the patients belonged to the districts of Jalandhar (5%), Kapurthala (5%), Hoshiarpur (4%), Pathankot (3%), Ferozpur (2%) and Mansa (1%). Three patients, each belonging to the states of Jammu and Kashmir, Himachal Pradesh and Uttar Pradesh, were also included. A total of 255 (5 males and 250 females) clinically confirmed breast cancer patients and 255 age and gender matched normal healthy individuals were recruited in the present study between January 2013 and March 2017. Patients who had received chemotherapy, radiotherapy or blood transfusion before surger, or had previous history of any malignancy were excluded from the study. Controls were biologically unrelated to cancer patients and were from the same geographical region as the patients. Individuals in the control group had no family history of any cancer or any other chronic disease. This study was undertaken after approval by the institutional ethics committee of Guru Nanak Dev University, Amritsar, Punjab, India. After informed consent, as per tenets of the Declaration of Helsinki,a5 mL peripheral venous blood sample was collected in an EDTA vial from each subject.

### DNA isolation and screening of *BRCA1* variants

Genomic DNA was extracted from peripheral blood lymphocytes by a standard phenol-chloroform method (Adeli and Ogbonna, 1990). Four variants of *BRCA1*, c.190T>C (p.Cys64Arg), 1307delT, g.5331G>A (p.G1738R) and c.2612C>T (p.Pro871Leu) were screened by a PCR-RFLP method using published primer sequences (Gajalakshmi *et al.*, 2007; Karami and Mehdipour*,* 2013; Xu *et al.*, 2018).

The PCR assays were set up in a 15 μL reaction volume containing 50 ng of DNA, 1X *Taq* buffer with 1.5 mM MgCl2, 0.3 μL dNTPs mixture (Bangalore GeNei), 6 pmol of each primer (Sigma, St. Louis, MO, USA), 1U *Taq* DNA Polymerase (Bangalore GeNei). The PCR conditions were: an initial denaturation at 95 °C for 5 min, followed by 35 cycles with denaturation at 95 °C for 45 s, annealing at 58 °C for 30 s and extension at 72 °C for 45 s, and a final extension step at 72 °C for 10 min, run in a Mastercycler gradient (Eppendorf, Germany) machine. A negative control without template DNA was run in each reaction to check for contamination. The PCR products were analysed on 2% ethidium bromide-stained agarose gels. Amplified products were digested with the respective restriction enzymes following the manufacturer’s instructions (New England Biolabs, Beverly, MA), details of which are shown in Table 1. The restriction digestion reaction products were also analysed on 2.5% ethidium bromide-stained agarose gels. The genotype was assigned to each sample according to the size of the fragments obtained after digestion (Table 1). Genotyping was performed without knowing the status of the subjects.

**Table 1:** Details of analyzed *BRCA1* variants and restriction conditions used for genotyping.

S. No.	Variant	PCR product size (bp)	Restriction enzyme used	Digestion temperature	Fragment size (bp) after digestion	Alleles
1.	c.190T>C (p.Cys64Arg)	193 bp	*SnaB*I	37 °C	193	T
					124 and 69	C
2.	g. 5331G>A (p.G1738R)	233 bp	*BsaX*I	37 °C	112, 91 and 30	G
					233	A
3.	1307delT	151 bp	*Dde*I	37 °C	151	WT
					96 and 55	MUT
4.	c.2612C>T (p.Pro871Leu)	125 bp	*Hpa*II	37 °C	99 and 26	C
					125	T

WT - wild type; MUT - mutant.

### Statistical analysis

A chi-square test was used to test for Hardy-Weinberg Equilibrium (HWE) and difference of genotype and allele frequencies between the breast cancer patients and normal controls. For determining the association between *BRCA1* variants with breast cancer risk, the odds ratio (OR) with 95% confidence interval (CI) was calculated. A genetic model analysis was done to check for an association of the BRCA1 variants with breast cancer risk. A value of p ≤ 0.05 was considered statistically significant. Statistical analysis was performed by using SPSS (Version 20.0; SPSS, Inc).

## Results

The present study recruited 255 clinically and histopathologically confirmed breast cancer patients and 255 healthy controls. Out of the 255 patients, 250 (98%) were females and 5 (2%) were males. The mean age of the patients was 50.7 ± 11.43 years (range 27-85 years) and that of controls was 48.56 ± 14.26 (range 17-90 years). The frequency of patients diagnosed with stage I, stage II, stage III and stage IV breast cancer were 14.9%, 46.8%, 25.5% and 12.8%, respectively (Table 2). Out of these, 40% of the patients were triple negative for the receptor status. The majority of the patients had sporadic breast cancer (78.8%), while 7.5% reported a history of breast cancer in their family (Table 3). Another 13.7% of the patients had a history of cancer of other sites. Hence, 21.2% of the patients had a family history of any type of cancer.

**Table 2:** Clinical profile of breast cancer patients.

Parameter	Total n (%)	Females n (%)	Males n (%)
Stage			
I	35(14.9)	35(15.2)	0(0)
II	110(46.8)	105(46.7)	5(100)
III	60(25.5	60(26.1)	0(0)
IV	30(12.8)	30(13)	0(0)
Breast side			
Left	116(47.2)	113(46.9)	3(60)
Right	121(49.2)	120(49.8)	1(20)
Bilateral	9(3.6)	8(3.3)	1(20)
Histological Type			
DCIS	6(2.7)	6(2.8)	0(0)
DCIS-Papilary	3(1.3)	3(1.4)	0(00
IDC	205(91.9)	200(91.7)	5(100)
IDC-Mucinous	5(2.2)	5(2.2)	0(0)
IDC-Medulllary	1(0.5)	1(0.5)	0(0)
IDC-Pagets	1(0.5)	1(0.5)	0(0)
Invasive Lobular Carcinoma	2(0.9)	2(0.9)	0(0)

n: number of subjects

**Table 3:** Breast cancer patients with family history of cancer.

Category	Cases	% of Total Cases
Sporadic cases	201	78.8
Familial cases	54	21.2
History of breast cancer		
1^st^ Relatives	11	4.3
2^nd^ Relatives	5	2.0
3^rd^ Relatives	3	1.2
History of other cancer		
1^st^ Relatives	23	9.0
2^nd^ Relatives	12	4.7
3^rd^ Relatives	0	0

The genotype and allele frequencies of the four analysed *BRCA1* variants in cases and controls are shown in Table 4. For the 3 variants c.190T>C (p.Cys64Arg), 1307delT and c.5331G>A (p.G1738R) all the patients and controls had the wild-type genotype (Figures S1-S4). The observed genotype frequencies for the c.2612C>T polymorphism were in HWE in the controls (*p*=0.11). The frequency of the CC, CT and TT genotypes of c.2612C>T polymorphism were 14.5% vs. 15.7%, 59.6% vs. 53.7% and 25.9% vs 30.6%, respectively, in breast cancer patients and control individuals. No association was observed between the breast cancer risk and the CT (OR = 1.19, 95% CI: 0.73-1.98, *p*=0.48) and TT (OR = 0.91, 95% CI:0.53-1.59, *p*=0.75) genotypes. The c.2612 C>T polymorphism did not show an association with breast cancer in any of the genetic models (Table 4). The T allele frequency was higher than that of the C allele in both patients and controls, but the difference between patients and controls was not significant (*p*=0.57). The difference in the frequency distribution of the C and T alleles in breast cancer patients with involvement of left, right or bilateral sides was statistically non-significant (*p*=0.55). This difference was also not significant (*p*=0.60) amongst patients at different stages of breast cancer.

**Table 4:** Association analyses of *BRCA1* variants with breast cancer risk.

Variant	Genotype	Patients n (%)	Controls n (%)	OR (95%CI)	*p*-value
c.190T>C (p.Cys64Arg)	TT	255(100)	255(100)	-	NC
	TC	0	0		
	CC	0	0		
1307delT	WT/WT	255(100)	255(100)	-	NC
	WT/MUT	0	0		
	MUT/MUT	0	0		
c.5331G>A (p.G1738R)	GG	255(100)	255(100)	-	NC
	GA	0	0		
	AA	0	0		
c.2612 C>T (p.Pro871Leu)	CC	37(14.5)	40(15.7)	1 (ref)	
	CT	152(59.6)	137(53.7)	1.19(0.73-1.98)	0.48
	TT	66(25.9)	78(30.6)	0.91(0.53-1.59)	0.75
	Allele				
	C	226(44.3)	217(42.5)	1 (ref)	
	T	284(55.7)	293(57.5)	0.93(0.73-1.19)	0.57
Genetic Models					
Dominant Model CT/TT vs CC				1.10(0.67-1.78)	0.71
Co-dominant Model TT vs CT =CT vs CC				0.92(0.70-1.20)	0.54
Recessive Model TT vs CC/CT				0.79(0.54-1.17)	0.24

n: number of subjects; OR: odds ratio; CI: confidence intervals; NC: not calculated.

To evaluate the relation between the parameters like age at diagnosis, menopausal history, habitat, diet and BMI with the *BRCA1* p.Pro871Leu variant, the cases and controls were stratified according to the aforementioned parameters. We found no significant difference in the genotypic frequencies among patients and controls. However, there was a significant difference in C and T allele frequencies among cases and controls for parameters like age at diagnosis in the group 36-50 years (OR = 0.47, 95% CI = 0.32-0.70, *p*=0.00), frequency of premenopausal women (OR = 0.56, 95% CI = 0.36-0.87, *p*=0.01), frequency of post-menopausal women (OR = 0.65, 95% CI = 0.46-0.90, p=0.01), rural habitat (OR = 0.51, 95% CI = 0.37-0.87, *p*=0.000), vegetarian diet (OR = 0.61, 95% CI = 0.44-0.85, *p*=0.003) and obesity (BMI ≥ 23 kg/m2) (OR = 0.62, 95% CI = 0.46-0.84, *p*=0.002) (Table 5).

**Table 5:** Correlation of *BRCA1* p.Pro871Leu variant with demographic parameters in subjects.

Parameter	Genotypes				OR (95% CI)	*p*-value	Allele				OR (95% CI)	*p*-value
	Cases		Controls				Cases		Controls			
	CC N (%)	CT+TT N (%)	CC N (%)	CT+TT N (%)			C N (%)	T N (%)	C N (%)	T N (%)		
Age at diagnosis (years)												
≤ 35	5(13.9)	26(10.9)	3(11.1)	45(20)	0.35(0.08-1.57)	0.17	26(11.7)	36(12.7)	34(19.9)	62(18.6)	0.76(0.39-1.46)	0.41
36-50	15(41.7)	118(49.6)	8(29.6)	96(42.7)	0.66(0.27-1.61)	0.36	106(47.5)	118(41.7)	62(36.3)	146(43.9)	0.47(0.32-0.70)	**0.00**
51-65	13(36.1)	77(32.4)	6(22.2)	66(29.3)	0.54(0.19-1.5)	0.24	73(32.7)	107(37.8)	45(26.3)	99(29.7)	0.67(0.42-1.06)	0.08
≥ 66	3(8.3)	17(7.1)	10(37.1)	18(8.0)	3.15(0.74-13.45)	0.12	18(8.1)	22(7.8)	30(17.5)	26(7.8)	1.41(0.62-3.19)	0.41
Menstrual history												
premenopausal	12(35.3)	66(32.0)	6(26.1)	88(42.3)	0.38(0.13-1.05)	0.06	71(33.3)	85(31.8)	60(39.5)	128(41.3)	0.56(0.36-0.87)	**0.01**
postmenopausal	22(64.7)	140(68.0)	17(73.9)	120(57.7)	1.46(0.56-3.18)	0.76	142(66.7)	182(68.2)	92(60.5)	182(58.7)	0.65(0.46-0.90)	**0.01**
Habitat												
rural	27(77.1)	163(76.9)	16(61.5)	162(73)	0.60(0.31-1.15)	0.12	167(77.0)	213(76.9)	101(60.8)	255(77.3)	0.51(0.37-0.69)	**0.000**
urban	8(22.9)	49(23.1)	10(38.5)	60(27)	1.02(0.37-2.78)	0.97	50(23.0)	64(23.1)	65(39.2)	75(22.7)	0.68(0.67-1.82)	0.68
Diet												
vegetarian	24(68.6)	136(64.5)	16(61.5)	134(61.5)	0.68(0.34-1.33)	0.26	146(67.3)	174(63.3)	102(61.8)	198(61.3)	0.61(0.44-0.85)	**0.003**
non-vegetarian	11(31.4)	75(35.5)	10(38.5)	84(38.5)	0.81(0.33-2.02)	0.65	71(32.7)	101(36.7)	63(38.2)	125(38.7)	0.72(0.47-1.10)	0.13
BMI												
non-obese	7(20.6)	60(29.9)	5(19.2)	42(19.0)	1.02(0.30-3.43)	0.97	56(26.9)	78(29.8)	30(18.1)	64(19.5)	0.65(0.38-1.14)	0.13
obese	27(79.4)	141(70.1)	21(80.8)	179(81.0)	0.61(0.33-1.23)	0.12	152(73.1)	184(70.2)	136(81.9)	264(80.5)	0.62(0.46-0.84)	**0.002**

n: number of subjects; OR: odds ratio; CI: confidence intervals; NC: not calculated; *p*<0.05 (significance).

## Discussion

In the present case-control study, we assessed the relationship of c.190T>C (p.Cys64Arg), 1307delT, g.5331G>A (p.G1738R) and c.2612C>T variants of *BRCA1* with breast cancer risk. The variants p.Cys64Arg and p.G1738R have never been reported from any Indian study so far, and also c.2612C>T had not yet been reported from India at the time when the present study was undertaken. It has been only recently reported from a population of West India (Gujarat), where 32 polymorphisms and two novel mutations in *BRCA1* were found in 35 breast cancer patients by an NGS method (Shah *et al.*, 2018). Among these, c.2612C>T was found in six out of the 35 patients.

The c.190T>C variant has been reported as a pathological mutation in families with HBOC (history of breast and ovarian cancer) in Polish (Jakubowska *et al.*, 2001), Italian (Willems *et al.*, 2009; Caleca *et al.*, 2014), Brazilian (Fernandes *et al.*, 2016), French (Azzollini *et al.*, 2017) and Japanese (Arai *et al.*, 2018) populations. A few *in-vitro* studies have also revealed its deleterious effects (Cochran *et al.*, 2015; Anantha *et al.*, 2017). In the present study, only the wild type genotype (TT) of c. 190T>C was found in all the patients and controls from Amritsar.

The 1307delT variant was first time reported in a South Indian family with Dravidian ancestry having breast cancer (Gajalakshmi *et al.*, 2007). The South Indian population represents a racial compositon different from that of North India. In the present study all the patients and controls, which were from the Northwest part of India, with Caucasian and Indoscythian ancestry (Reich *et al.*, 2009), had the wild type genotype for this variant.

The g.5331G>A (p.G1738R) missense mutation has been repeatedly found in Greek families with breast cancer (Belogianni *et al.*, 2004; Armaou *et al.*, 2009; Koumpis *et al.*, 2011), ovarian cancer (Stavropoulou *et al.*, 2013) or both (Konstantopoulou *et al.*, 2008; Karami and Mehdipour, 2013). It is also reported to be a deleterious mutation in *in-vitro* assays (Carvalho *et al.*, 2007; Chenevix-Trench *et al.*, 2016). None of the patients or controls in the present study carried this mutation.

The c.2612C>T (p.Pro871Leu) non-synonymous SNP causes an amino acid change from proline to leucine at position 871 in the BRCA1 protein. The previous published studies give contradictory reports on the role of p.P871L and the risk for various types of cancers. The wild type genotype (CC) was associated with increased risk of gastric cancer (Wang *et al.*, 2015), esophageal squamous cell carcinoma (ESCC) (Zhang *et al.*, 2013) and non-Hodgkin Lymphoma (NHL) (Chen *et al.*, 2013), but not associated with thyroid carcinoma (Xu *et al.*, 2012) and ovarian cancer (Wenham *et al.*, 2003; Auranen *et al.*, 2005). In a recent meta-analysis (Xu *et al.*, 2018), the p.Pro871Leu polymorphism was linked to decreased risk of various cancers like ESCC, cervical cancer, gastric cancer and NHL among Asians. But some studies from Chinese populations have reported significant associations between the *BRCA1* p.Pro871Leu variant and breast cancer, where the CT genotype provides increased risk to breast cancer in these populations (Huo *et al.*, 2009; Wang *et al.*, 2009). The homozygous variant TT genotype has also been reported to be associated with reduction in glioblastoma risk in Caucasians (Chang *et al.*, 2008) and NHL risk in Korea (Kim *et al.*, 2014). The T allele of the p.Pro871Leu polymorphism has been shown to be linked with miR-628 dependent BRCA1 reduction, indicating that cells with the T allele would express higher BRCA1 as compared to those with the C allele (Nicoloso *et al.*, 2010). In the present study, none of the genotypes of this variant were found to be associated with breast cancer risk. Similar findings have been reported in a Czech population (Soucek *et al.*, 2007), where no association was reported. Meta-analyses carried out in 2014 (Qin *et al.*, 2014) and 2017 (Miao *et al.*, 2017) reported no association of the p.Pro871Leu polymorphism and breast cancer risk. Contradictory results have also been reported for the *BRCA1* 871L allele, as significantly associated with increased risk of breast cancer in a Polish population (Smolarz *et al.*, 2017). An association of the TT genotype with reduced breast cancer risk in Chinese has also been reported (Zhou *et al.*, 2009).

When assessed for response to therapy, p.Pro871Leu showed no association with either advanced phases of CML or treatment response (Gutierrez-Malacatt *et al.*, 2016). The CT/TT genotypes have been reported to be associated with ovarian cancer progression in non-African American patients, but not with progression-free survival (PFS) and overall survival (OS) in patients treated with cisplatin and paclitaxel (Tian *et al.*, 2013). The TT genotype has been reported to not only contribute to increased breast cancer risk associated with combined estrogen monotherapy in postmenopausal women in Germany (Abbas *et al.*, 2010), but also with recurrence of TNBC after radiotherapy in Han Chinese (Shi *et al.*, 2017).

Understanding of the biological mechanisms underlying breast cancer predisposition by genomic profiling can help to define molecular targets for chemoprevention and biomarkers of breast cancer risk. India is the second most populous country in the world, but not much information on *BRCA1* is available as of now. The 185delAG variant has been found to be the most common mutation in BRCA1 in India, as revealed by a few reported studies of limited sample sizes (range 19 - 204). It has been found in variable frequencies in the studied groups from North India: 7.14% (Kumar *et al.*, 2002), 0.49% (Saxena *et al.*, 2006) and South India: 7 out of 19 members in a single family study (Kadalmani *et al.*, 2007), 54.5% (Vinodkumar *et al.*, 2007) and 16.4% (Vaidyanathan *et al.*, 2009). The present study is the first report providing baseline data on four variants of *BRCA1* from the Amritsar and adjoining regions of Punjab, Northwest India. The three mutations [c.190T>C (p.Cys64Arg), 1307delT and g.5331G>A (p.G1738R)], which have been widely known as pathogenic in Caucasians, have no mutant genotype in the studied population. For the c.2612C>T polymorphism also, the T allele frequency was slightly higher than that of the C allele, and there was a preponderance of the CT genotype, but no association with the breast cancer risk. A marked variation in the distribution of p.Pro871Leu in the Northwest Indian population from the previously reported populations of South India has been observed. The present study indicates the need of screening Indian populations for other mutations/variants in *BRCA1* gene for identifying breast cancer risk and possible targets for therapy.

## Supplementary material

The following online material is available for this article:

Figure S1 - Agarose gel picture for BRCA1 c.190T>C (p.Cys64Arg).

Figure S2 - Agarose gel picture for BRCA1 1307delT.

Figure S3 - Agarose gel picture for BRCA1 c.5331G>A (p.G1738R).

Figure S4 - Agarose gel picture for BRCA1 c.2612 C>T (p.Pro871Leu).

## References

[B1] Abbas S, Beckmann L, Changclaude J, Hein R, Kropp S, Parthimos M, Dunnebier T, Hamann U, Brors B, Eils R (2010). MARIE-GENICA Consortium on genetic susceptibility for menopausal
hormone therapy related breast cancer risk polymorphisms in the BRCA1 and
ABCB1 genes modulate menopausal hormone therapy associated breast cancer
risk in postmenopausal women. Breast Cancer Res Treat.

[B2] Adeli K, Ogbonna G (1990). Rapid purification of human DNA from whole blood for potential
application in clinical chemistry laboratories. Clin Chem.

[B3] Anantha RW, Simhadri S, Foo TK, Miao S, Liu J, Shen Z, Ganesan S, Xia B (2017). Functional and mutational landscapes of BRCA1 for
homology-directed repair and therapy resistance. eLife.

[B4] Arai M, Yokoyama S, Watanabe C, Yoshida R, Kita M, Okawa M, Sakurai A, Sekine M, Yotsumoto J, Nomura H (2018). Genetic and clinical characteristics in Japanese hereditary
breast and ovarian cancer: First report after establishment of HBOC
registration system in Japan. J Hum Genet.

[B5] Armaou S, Pertesi M, Fostira F, Thodi G, Athanasopoulos PS, Kamakari S, Athanasiou A, Gogas H, Yannoukakos D, Fountzilas G (2009). Contribution of BRCA1 germ-line mutations to breast cancer in
Greece: A hospital-based study of 987 unselected breast cancer
cases. Br J Cancer.

[B6] Auranen A, Song H, Waterfall C, DiCioccio RA, Kuschel B, Kjaer SK, Hogdall E, Hogdall C, Stratton J, Whittemore AS (2005). Polymorphisms in DNA repair genes and epithelial ovarian cancer
risk. Int J Cancer.

[B7] Azzollini J, Pesenti C, Ferrari L, Fontana L, Calvello M, Peissel B, Portera G, Tabano S, Carcangiu ML, Riva P (2017). Revertant mosaicism for family mutations is not observed in
BRCA1/2 phenocopies. PLoS One.

[B8] Batra APS, Sidhu S, Sambyal V (2010). Obesity among breast and esophagus cancer patients of Amritsar
district, Punjab. Anthropologist.

[B9] Belogianni I, Apessos A, Mihalatos M, Razi E, Labropoulos S, Petounis A, Gaki V, Keramopoulos A, Pandis N, Kyriacou K (2004). Characterization of a novel large deletion and single point
mutations in the BRCA1 gene in a Greek cohort of families with suspected
hereditary breast cancer. BMC Cancer.

[B10] Bhasin MK, Walter H, Danker-Hopfe H (1992). The distribution of genetical, morphological and behavioural traits
among the peoples of Indian region.

[B11] Caleca L, Putignano AL, Colombo M, Congregati C, Sarkar M, Magliery TJ, Ripamonti CB, Foglia C, Peissel B, Zaffaroni D (2014). Characterization of an Italian founder mutation in the
RING-finger domain of BRCA1. PLoS One.

[B12] Carvalho MA, Marsillac SM, Karchin R, Manoukian S, Grist S, Swaby RF, Urmenyi TP, Rondinelli E, Silva R, Gayol L (2007). Determination of cancer risk associated with germ line BRCA1
missense variants by functional analysis. Cancer Res.

[B13] Chang JS, Yeh RF, Wiencke JK, Wiemels JL, Smirnov I, Pico AR, Tihan T, Patoka J, Miike R, Sison JD (2008). Pathway analysis of single-nucleotide polymorphisms potentially
associated with glioblastoma multiforme susceptibility using random
forests. Cancer Epidemiol Biomarkers Prev.

[B14] Chen Y, Zheng T, Lan Q, Kim C, Qin Q, Foss F, Chen X, Holford T, Leaderer B, Boyle P (2013). Polymorphisms in DNA repair pathway genes, body mass index, and
risk of non-Hodgkin lymphoma. Am J Hematol.

[B15] Chenevix-Trench G, Healey S, Lakhani S, Waring P, Cummings M, Brinkworth R, Deffenbaugh AM, Burbidge LA, Pruss D, Judkins T (2006). Genetic and histopathologic evaluation of BRCA1 and BRCA2 DNA
sequence variants of unknown clinical significance. Cancer Res.

[B16] Clark SL, Rodriguez AM, Snyder RR, Hankins GD, Boehning D (2012). Structure-function of the tumor suppressor BRCA. Comput Struct Biotechnol J.

[B17] Cochran RL, Cidado J, Kim M, Zabransky DJ, Croessmann S, Chu D, Wong HY, Beaver JA, Cravero K, Erlanger B (2015). Functional isogenic modeling of BRCA1 alleles reveals distinct
carrier phenotypes. Oncotarget.

[B18] Fernandes GC, Michelli RA, Galvao HC, Paula AE, Pereira R, Andrade CE, Felicio PS, Souza CP, Mendes DR, Volc S (2016). Prevalence of BRCA1/BRCA2 mutations in a Brazilian population
sample at-risk for hereditary breast cancer and characterization of its
genetic ancestry. Oncotarget.

[B19] Gajalakshmi P, Natarajan TG, Rani DS, Thangaraj K (2007). A novel BRCA1 mutation in an Indian family with hereditary
breast/ovarian cancer. Breast Cancer Res Treat.

[B20] Ghoncheh M, Pournamdar Z, Salehiniya H (2016). Incidence and mortality and epidemiology of breast cancer in the
world. Asian Pacif J Cancer Prev.

[B21] Glover JN (2006). Insights into the molecular basis of human hereditary breast
cancer from studies of the BRCA1 BRCT domain. Fam Cancer.

[B22] Gutierrez-Malacatt H, Ayala-Sanchez M, Aquino-Ortega X, Dominguez-Rodriguez J, Martinez-Tovar A, Martinez-Hernandez A, Contreras-Cubas CC, Orozco L, Cordova EJ (2016). The rs61764370 functional variant in the KRAS oncogene is
associated with chronic myeloid leukemia risk in women. Asian Pac J Cancer Prev.

[B23] Huo X, Lu C, Huang X, Hu Z, Jin G, Ma H, Wang X, Qin J, Wang X, Shen H (2009). Polymorphisms in BRCA1, BRCA1-interacting genes and
susceptibility of breast cancer in Chinese women. J Cancer Res Clin Oncol.

[B24] Jakubowska A, Gorski B, Byrski T, Huzarski T, Gronwald J, Menkiszak J, Cybulski C, Debniak T, Hadaczek P, Scott RJ (2001). Detection of germline mutations in the BRCA1 gene by RNA-based
sequencing. Hum Mutat.

[B25] Kadalmani K, Deepa S, Bagavathi S, Anishetty S, Thangaraj K, Gajalakshmi P (2007). Independent origin of 185delAG BRCA1 mutation in an Indian
family. Neoplasma.

[B26] Karami F, Mehdipour PA (2013). Comprehensive focus on global spectrum of BRCA1 and BRCA2
mutations in breast cancer. Biomed Res Int.

[B27] Karchin R, Monteiro AN, Tavtigian SV, Carvalho MA, Sali A (2007). Functional impact of missense variants in BRCA1 predicted by
supervised learning. PLoS Comput Biol.

[B28] Kim H, Choi DH (2013). Distribution of BRCA1 and BRCA2 mutations in Asian patients with
breast cancer. J Breast Cancer.

[B29] Kim HN, Kim NY, Yu L, Kim YK, Lee IK, Yang DH, Lee JJ, Shin MH, Park KS, Choi JS (2014). Polymorphisms in DNA repair genes and MDR1 and the risk for
non-Hodgkin lymphoma. Intl J Mol Sci.

[B30] Konstantopoulou I, Rampias T, Ladopoulou A, Koutsodontis G, Armaou S, Anagnostopoulos T, Nikolopoulos G, Kamakari S, Nounesis G, Stylianakis A (2008). Greek BRCA1 and BRCA2 mutation spectrum: two BRCA1 mutations
account for half the carriers found among high-risk breast/ovarian cancer
patients. Breast Cancer Res Treat.

[B31] Koumpis C, Dimitrakakis C, Antsaklis A, Royer R, Zhang S, Narod SA, Kotsopoulos J (2011). Prevalence of BRCA1 and BRCA2 mutations in unselected breast
cancer patients from Greece. Hered Cancer Clin Pract.

[B32] Kumar BV, Lakhotia S, Ankathil R, Madhavan J, Jayaprakash PG, Nair MK, Somasundaram K (2002). Germline BRCA1 mutation analysis in Indian breast/ovarian cancer
families. Cancer Biol Ther.

[B33] Miao L, Yu Y, Ji Y, Zhang B, Yuan Z, Du Y, Zhu L, Wang R, Chen N, Yuan H (2017). Association between BRCA1 P871L polymorphism and cancer risk:
evidence from a meta-analysis. Oncotarget.

[B34] Miki Y, Swensen J, Shattuck-Eidens D, Futreal PA, Harshman K, Tavtigian S (1994). A strong candidate for the breast and ovarian cancer
susceptibility gene BRCA1. Science.

[B35] Naga AP (2011). Epidemiology of genetic alterations in progression of breast
cancer. J Carcinog Mutagen.

[B36] Nicoloso MS, Sun H, Spizzo R, Kim H, Wickramasinghe P, Shimizu M, Wojcik SE, Ferdin J, Kunej T, Xiao L (2010). Single-nucleotide polymorphisms inside microRNA target sites
influence tumor susceptibility. Cancer Res.

[B37] Parvin JD (2004). Overview of history and progress in BRCA1 research: The first
BRCA1 decade. Cancer Biol Ther.

[B38] Qin TT, Chen T, Zhang Q, Du HN, Shu YQ, Luo K, Zhu LJ (2014). Association between BRCA1 rs799917polymorphism and breast cancer
risk: A meta-analysis of 19,878 subjects. Biomed Pharmacother.

[B39] Reich D, Thangaraj K, Patterson N, Price AL, Singh L (2009). Reconstructing Indian population history. Nature.

[B40] Saxena S, Chakraborty A, Kaushal M, Kotwal S, Bhatanager D, Mohil RS, Chintamani C, Aggarwal AK, Sharma VK, Sharma PC (2006). Contribution of germline BRCA1 and BRCA2 sequence alterations to
breast cancer in Northern India. BMC Med Genet.

[B41] Shah ND, Shah PS, Panchal YY, Katudia KH, Khatri NB, Ray HSP, Bhatiya UR, Shah SC, Shah BS, Rao MV (2018). Mutation analysis of BRCA1/2 mutations with special reference to
polymorphic SNPs in Indian breast cancer patients. Appl Clin Genet.

[B42] Shi M, Ma F, Liu J, Xing H, Zhu H, Yu J, Yang M (2017). A functional BRCA1 coding sequence genetic variant contributes to
prognosis of triple-negative breast cancer, especially after
radiotherapy. Breast Cancer Res Treat.

[B43] Smolarz B, Brys M, Forma E, Zadrozny M, Bienkiewicz J, Romanowicz H (2017). Data on single nucleotide polymorphism of DNA repair genes and
breast cancer risk from Poland. Pathol Oncol Res.

[B44] Soucek P, Borovanova T, Pohlreich P, Kleibl Z, Novotny J (2007). Role of single nucleotide polymorphisms and haplotypes in BRCA1
in breast cancer: Czech case–control study. Breast Cancer Res Treat.

[B45] Stavropoulou AV, Fostira F, Pertesi M, Tsitlaidou M, Voutsinas GE, Triantafyllidou O, Bamias A, Dimopoulos MA, Timotheadou E, Pectasides D (2013). Prevalence of BRCA1 mutations in familial and sporadic greek
ovarian cancer cases. PLoS One.

[B46] Tian C, Darcy KM, Krivak TC, DeLoia JA, Armstrong D, Davis W, Zhao H, Moysich K, Ambrosone CB (2013). Assessment of the prognostic value of two common variants of
BRCA1 and BRCA2 genes in ovarian cancer patients treated with cisplatin and
paclitaxel: a Gynecologic Oncology Group study. Front Oncol.

[B47] Vaidyanathan K, Lakhotia S, Ravishankar HM, Tabassum U, Mukherjee G, Somasundaram K (2009). BRCA1 and BRCA2 germline mutation analysis among Indian women
from South India: identification of four novel mutations and high-frequency
occurrence of 185delAG mutation. J Biosci.

[B48] Vinodkumar B, Syamala V, Abraham EK, Balakrishnan R, Ankathil R (2007). Germline BRCA1 mutation and survival analysis in familial breast
cancer patients in Kerala, South India. J Exp Clin Cancer Res.

[B49] Wang K, Xu L, Pan L, Xu K, Li G (2015). The functional BRCA1 rs799917 genetic polymorphism is associated
with gastric cancer risk in a Chinese Han population. Tumour Biol.

[B50] Wang Z, Xu Y, Tang J, Ma H, Qin J, Lu C, Wang X, Hu Z, Wang X, Shen H (2009). A polymorphism in Werner syndrome gene is associated with breast
cancer susceptibility in Chinese women. Breast Cancer Res Treat.

[B51] Wenham RM, Schildkraut JM, McLean K, Calingaert B, Bentley RC, Marks J, Berchuck A (2003). Polymorphisms in BRCA1 and BRCA2 and risk of epithelial ovarian
cancer. Clin Cancer Res.

[B52] Willems P, Magri V, Cretnik M, Fasano M, Jakubowska A, Levanat S, Lubinski J, Marras E, Musani V, Thierens H (2009). Characterization of the c. Int J Oncol.

[B53] Williams RS, Chasman DI, Hau DD, Hui B, Lau AY, Glover JM (2003). Detection of protein folding defects caused by BRCA1-BRCT
truncation and missense mutations. J Biol Chem.

[B54] Xu L, Doan PC, Wei Q, Liu Y, Li G, Sturgis EM (2012). Association of BRCA1 functional single nucleotide polymorphisms
with risk of differentiated thyroid carcinoma. Thyroid.

[B55] Xu GP, Zhao Q, Wang D, Xie WY, Zhou H, Chen SZ, Wu LF (2018). The association between BRCA1 gene polymorphism and cancer risk:
a meta-analysis. Oncotarget.

[B56] Zhang X, Wei J, Zhou L, Zhou C, Shi J, Yuan Q, Yang M, Lin D (2013). A functional BRCA1 coding sequence genetic variant contributes to
risk of esophageal squamous cell carcinoma. Carcinogenesis.

[B57] Zhou X, Han S, Wang S, Chen X, Dong J, Shi X, Xia Y, Wang X, Hu Z, Shen H (2009). Polymorphisms in HPV E6/E7 protein interacted genes and risk of
cervical cancer in Chinese women: a case-control analysis. Gynecol Oncol.

