# Supplementary material for: Screening of BRCA1 variants c.190T>C, 1307delT, g.5331G>A and c.2612C>T in breast cancer patients from North India
Source: Genet Mol Biol. 2020 May 20;43(2):e20190014. doi: 10.1590/1678-4685-GMB-2019-0014 (PMC7250277; doi:10.1590/1678-4685-GMB-2019-0014)
Supplement: Figure S4 [file 1415-4757-GMB-43-2-e20190014-s4.pdf]

Supplementary material to: Screening of *BRCA1* variants c.190T>C, 1307delT, g.5331G>A and c.2612C>T in breast cancer patients from North India

BRCA1 c.2612 C>T (p.Pro871Leu)

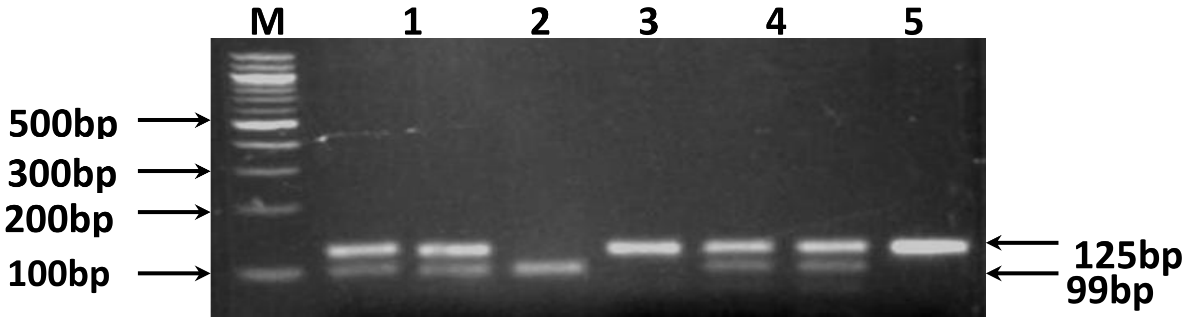

Figure S4- A photograph of 2.5% agarose gel stained with ethidium bromide showing restriction digestion products. M represents 100 bp Molecular Weight Marker; Lane 3 CC genotype; Lane 1, 2, 5, 6 CT genotype; Lane 4 TT genotype; UD Undigested Product. Band of 26 bp is not visible on the gel picture.
